# Antibodies directed to antigens secreted by murine epithelioid macrophages modulate BCG-induced granulomata

**DOI:** 10.1155/S0962935193000316

**Published:** 1993

**Authors:** Mario Mariano, Tania Aguiar, Celia R. W. Carneiro, Edilberto Postol, Venânclo A. F. Alves, Ricardo Ribeiro-dos-Santos

**Affiliations:** 1 Department of Immunology Institute of Biomedical Sciences University of São Paulo SP Brazil; 2 Ludwig Institute for Cancer Research São Paulo branch São Paulo SP Brazil; 3 Department of Pathology Adolpho Lutz Institute SP Brazil; 4 Department of Biochemistry and Molecular Biology Oswaldo Cruz Institute Rio de Janeiro RJ Brazil

## Abstract

The authors have previously shown that epithelioid cells isolated from mice secrete a factor, called macrophage deactivating factor (MDF), that promptly deactivates superoxide release by activated macrophages and neutrophils. In this paper some biological properties of a polyclonal rat antiserum directed to MDF and other substances secreted by these cells are described. The immunoglobulin fraction of this antiserum reacted, by immunocytochemical methods, with epitopes in the cell membrane of macrophages adherent to coverslips subcutaneously implanted for 14 days; but not for 5 days. It also reacted with antigens within and outside cells in BCG-induced granulomas. This antiserum blocked completely the macrophage deactivating activity of epithelioid cell culture supernatants. Anti-IL-10 monoclonal antibody, did not block MDF activity. The administration of the immunoglobulin fraction from immunized rats to C_5_ deficient mice bearing BCG-induced granulomatas in the footpad, significantly reduced the size of the lesions. A marked necrosis of inflammatory cells and mononuclear cells phagocyting debris of necrotic cells were observed in these lesions.


Mediators of Inflammation 2, 229–233 (1993)

### Antibodies directed to antigens secreted by murine epithelioid macrophages modulate BCG-induced granulomata

Mario Mariano,[1,CA] Tania Aguiar,[1] Celia R. W. Carneiro,[2] Edilberto Postol[2] Venâncio A. F. Alves and Ricardo Ribeiro-dos-Santos[4]

[1]Department of Immunology, Institute of Biomedical Sciences, University of São Paulo, SP, Brazil; [2]Ludwig Institute for Cancer Research, São Paulo branch, São Paulo, SP, Brazil; [3]Department of Pathology, Adolpho Lutz Institute, SP, Brazil and [4]Department of Biochemistry and Molecular Biology, Oswaldo Cruz Institute, Rio de Janeiro, RJ, Brazil

[CA] Corresponding Author

**THE authors have previously shown that epithelioid cells isolated from mice secrete a factor, called macrophage deactivating factor (MDF), that promptly deactivates superoxide release by activated macrophages and neutrophils. In this paper some biological properties of a polyclonal rat antiserum directed to MDF and other substances secreted by these cells are described. The immunoglobulin fraction of this antiserum reacted, by immunocytochemical methods, with epitopes in the cell membrane of macrophages adherent to coverslips subcutaneously implanted for 14 days; but not for 5 days. It also reacted with antigens within and outside cells in BCG-induced granulomas. This antiserum blocked completely the macrophage deactivating activity of epithelioid cell culture supernatants. Anti-IL-10 monoclonal antibody, did not block MDF activity. The administration of the immunoglobulin fraction from immunized rats to $C_5$ deficient mice bearing BCG-induced granulomatas in the footpad, significantly reduced the size of the lesions. A marked necrosis of inflammatory cells and mononuclear cells phagocyting debris of necrotic cells were observed in these lesions.**

**Key words:** Epithelioid cells, Granuloma modulation, Macrophage deactivating factor (MDF)

### Introduction

Epithelioid cells are characteristic cells of granulomatous inflammation. Under a granulomatogenic stimulus, monocytes emigrate to tissues, become activated macrophages and differentiate or modulate into pre-epithelioid and epithelioid cells.[1–3] The role played by these cells in the maintenance and fate of this type of chronic inflammatory lesion is almost unknown. Epithelioid cells experimentally induced in mice are non-phagocytic and do not express Fc, C3b and nonspecific surface receptors.[4] The lack of receptor expression in these cells is not a permanent phenomenon since they can be re-expressed when they are cultured in levamisole containing medium.[5]

It has been suggested that secretion is the main function of epithelioid cells.[6,7] The secretory property of experimentally induced pre-epithelioid and epithelioid cells was first demonstrated by Camarero *et al.*[8] These cells secrete a factor named macrophage deactivating factor (MDF) which switches off superoxide anion release by activated macrophages and neutrophils. This factor was characterized as a heat stable, trypsin sensitive 11 kDa protein that interferes with membrane oxidase function of leukocytes.[9] Based on the *in vitro* properties of this factor, it was hypothesized that by deactivating newly arrived macrophages, MDF might play a fundamental role in the persistence of granulomatous lesions.[10]

In an attempt to further understand the role played by MDF and other epithelioid cell secreted substances on the maintenance and fate of infectious granulomata *in vivo*, a polyclonal antiserum directed to antigens secreted by these cells was made and its effects on the evolution of lesions induced by bacillus Clamette–Guérin (BCG) was investigated.

### Materials and Methods

*Animals:* Outbred mice from the Swiss strain or $C_5$ deficient mice from the A/J strain were used for the production of inflammatory cells. Male rats from the Wistar strain were used for intrasplenic immunization.

*Inflammatory cells:* Inflammatory cells were obtained by insertion of 12 mm round glass coverslips into the subcutaneous tissue of mice[11] and removed 5 and 14 days after implantation.

*Antigens:* Epithelioid cell secreted substances (ECSS), containing MDF,[8] were obtained as follows: coverslips were removed 14 days after implantation and kept in a 2 ml cuvette for 30 min, in phosphate buffered saline supplemented with 100 mM/ml of glucose, $Ca^{2+}$ and $Mg^{2+}$ (PBS-G)

© 1993 Rapid Communications of Oxford Ltd pH 7.2. The cuvette was built with neutral plastic and silicon sealer. Internal slits were made in the lateral walls to hold up to 15 coverslips in a vertical position. These conditioned media (CM) were pooled, filtered in a 0.2 $\mu$m Millipore filter and stored at $-20°C$. The CM were 10 times concentrated (CCM) in an ultrafiltration device (Amicon) with a cut-off of 10 kDa and stored at $-20°C$. Concentrated conditioned media were prepared with coverslips removed after 5 ($CCM_5$) or 14 ($CCM_{14}$) days of inflammation.

*Immunization schedule:* Rats were immunized by intrasplenic inoculation[12] of 0.1 ml of $CCM_{14}$. One week after intrasplenic injection, the animals were injected i.v. four times—1 week apart from each inoculation—with 1 ml of CM. One week after the last injection the animals, under ether anaesthesia, were bled by intracardiac puncture, the serum immunoglobulins purified by ammonium sulphate precipitation and concentrated twice (50 mg protein/ml) by dialysis against PBS. Immunoglobulins from normal rat serum were also prepared and concentrated as above.

*Immunocytochemistry:* Immunoglobulins from normal or immunized rats, were used to detect antigens in cells adherent to coverslips removed after 5 or 14 days. Cryostat sections of BCG induced lesions in the hind footpad of mice were also used. These preparations were fixed in cold methanol or acetone and submitted to immunocytochemical reactions using rabbit anti-IgG antiserum (Sigma). Controls included omission of the primary antibody and the use of irrelevant antibodies of the same IgG subclass on sections for the same period of incubations. Before immunostaining, sections were hydrated in buffer PBS (pH 7.4) and sequentially incubated with primary antibody, biotinilated goat anti-rat IgG (Vector, Burlingame, CA), followed by streptavidin–biotin conjugated to peroxidase (Amersham, UK) for 30 min, with 10 min PBS washes between each incubation. The reaction was developed with aminoethyl carbazole chromogen in the presence of hydrogen peroxide, counter-stained with Mayer's haematoxylin and mounted in glycerin–gelatin.

*Influence of ECSS and anti-ECSS on superoxide anion liberation by mouse activated macrophages:* Mice were inoculated i.p. with $5 \times 10^6$ viable BCG. Seven days later, macrophages were harvested by washing the peritoneal cavity of the animals with 3 ml of PBS. The cell suspension was washed twice in cold PBS before starting the assay. Cells were resuspended to achieve the concentration of $4 \times 10^6$ cells/ml in PBS-G. After centrifugation, cell pellets were resuspended, distributed into plastic tubes (0.5 ml/tube, $2 \times 10^6$ cells), the cells recentrifuged and resuspended in the following solutions: F-c plus 250 $\mu$l of PBS-G; F-c plus 200 $\mu$l of PBS-G and 50 $\mu$l of phorbol miristate acetate 1 ng/ml (PMA); F-c 200 $\mu$l of PBS-G, 50 $\mu$l of PMA, 125 $\mu$l of PBS-G, 125 $\mu$l $CCM_{14}$; F-c 200 $\mu$l of PBS-G, 50 $\mu$l of PMA and 125 $\mu$l of immunoglobulin (1:50) from immunized rats, 125 $\mu$l of $CCM_{14}$; F-c 200 $\mu$l of PBS-G, 50 $\mu$l of PMA and 125 $\mu$l of immunoglobulin (1 : 50) from normal rat serum, 125 $\mu$l of $CCM_{14}$. The following solution was taken as a blank: 250 $\mu$l of F-c, 4 mg in PBS-G (F-c) plus 250 $\mu$l of PBS-G without cells.

Immunoglobulins from normal and immunized rats (1 : 50) were mixed with equal volume of $CCM_{14}$, incubated for 1 h at 37°C and further incubated for 40 min with activated macrophages prior to addition to the reaction solutions. Four wells of a 96-well plate were filled with 100 $\mu$l of each of these cell suspensions and kept at 37°C for 1 h before reading in an ELISA reader (Dynatech MR 5000) with a 550 nm filter.[13]

*Influence of anti-IL-10 on MDF activity:* Anti-IL-10 monoclonal antibody produced by the SXC-2 hybridoma donated by DNAX Research Institute of Molecular and Cellular Biology Inc., Palo Alto, CA, USA, was used. Anti-IL-10 (10 $\mu$g of protein per ml) was added to $CCM_{14}$ at different concentrations and incubated for 1 h at 37°C. This solution was tested for MDF activity as above.

*Immunoglobulin administration:* Mice injected with BCG ($5 \times 10^6$ viable particles) in the hind footpad 14 days before, received four daily i.p. injections of 0.2 ml (10 mg protein) of concentrated immunoglobulins obtained from normal or immunized rats. The footpad thickness increase was daily measured as described above. Seven days after the first injection the animals were sacrificed, the BCG induced lesions removed, fixed in Bouin's fixative and processed for histological examination.

*Statistical analysis:* Means of results were compared by Student's *t*-test or two-way ANOVA followed by the Duncan test. A probability level of less than 0.05 was taken as significant.

### Results

*Anti-MDF antibodies are present in anti-ECSS antiserum:* The addition of CCM plus PMA to a suspension of BCG activated macrophages almost completely inhibited superoxide release by these cells. Conversely, the addition of immunoglobulins from immunized but not from normal rats to the system, restored the ability of activated macrophages to release superoxide anions, demonstrating that anti-MDF antibodies are present in immune serum (Fig. 1).

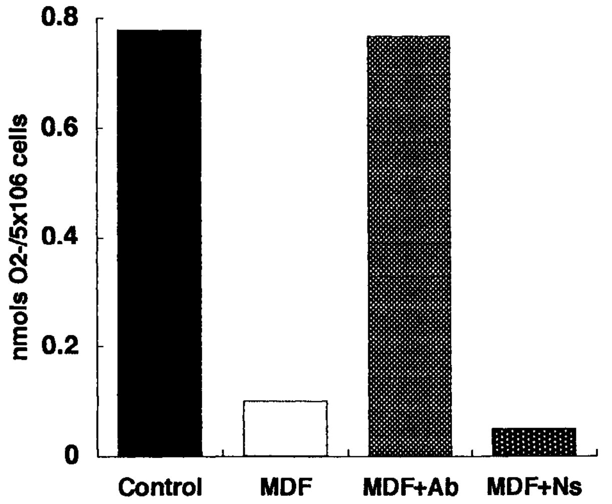

FIG. 1. The $CM_{14}$ blocks the liberation of superoxide anions by peritoneal mouse BCG-activated macrophages *in vitro*. The addition of anti-ECSS antibodies (Ab) reverts this activity and antibodies from normal rat serum (Ns) have no effect.

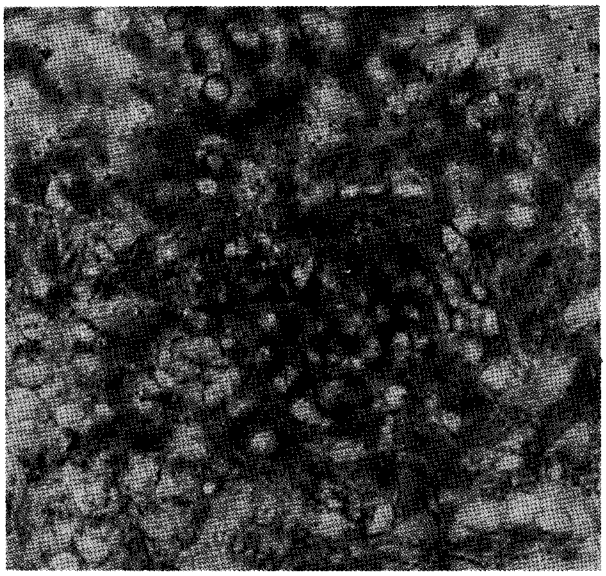

FIG. 3. Using the same technique as in Fig. 2 antibodies anti-ECSS detect epitopes in cells of the central area of BCG-induced granuloma evoked by the bacteria inoculation 14 days before (100×).

*MDF* vs *IL-10:* The addition of anti-IL-10 monoclonal antibody to CCM did not inhibit $O_2^-$ production by activated macrophages *in vitro*.

*Immunocytochemistry:* When anti-ECSS immunoglobulins were used as primary antibody (1:5 dilution), a strong staining was detected delimitating most of the cells obtained after 14 days of coverslip implantation (Fig. 2). The reaction was negative using cells obtained after 5 days of coverslip implantation. Negative results were found in the following controls: cells from 14 days of coverslip implantation submitted to the reaction using PBS or BSA instead of primary antiserum; using normal rat serum (1:200) instead of primary antiserum; omission of the secondary antibody incubation; omission of the amplification complex and reaction only with the substrates.

When cryostat sections of 14 day BCG induced granulomatas were tested as above, the reaction was positive within and outside cells mainly in the central area of the granulomatas (Fig. 3).

*Anti-ECSS immunoglobulins influence the evolution of BCG induced granulomatas:* The i.p. administration of 5 mg of purified immunoglobulins from immunized rats, for 4 consecutive days, significantly reduced the size of granulomatas induced by BCG injection 14 days before in the footpad of the animals. As shown in Fig. 4, the administration of immunoglobulins from normal rat serum did not influence the thickness of the lesions.

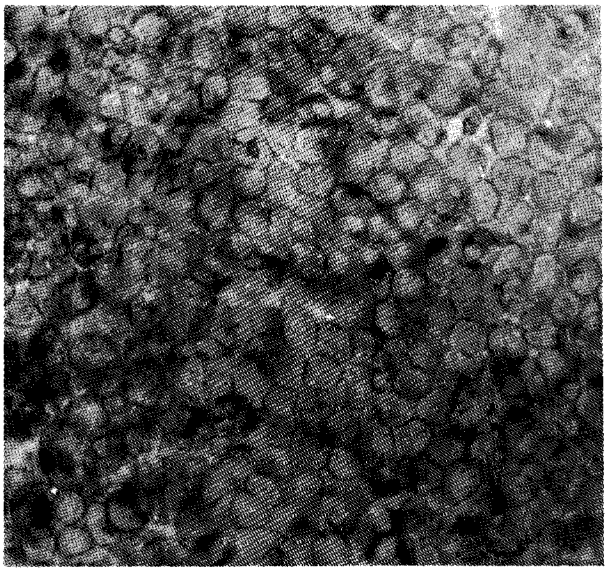

FIG. 2. Antibodies anti-ECSS detect epitopes in cells on the surface of coverslips removed after 14, but not after 5 days (not shown) of implantation in the subcutaneous tissue of mice. The antibodies are revealed by immunoperoxidase technique (100×).

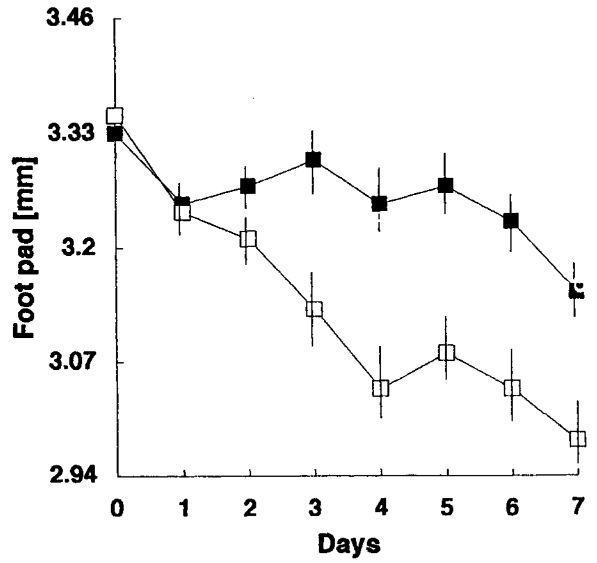

FIG. 4. The intraperitoneal injection, for four consecutive days, of anti-ECSS (□) to mice bearing BCG-induced granulomas in the footpad for 14 days, significantly reduced the size of the lesions after the third day of treatment ($p < 0.05$).

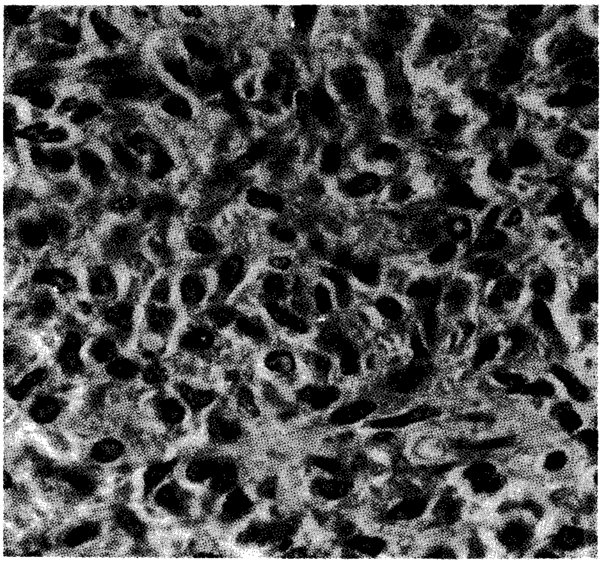

FIG. 5. Histopathology of typical arrangement of inflammatory macrophages in a granuloma induced by BCG inoculation 21 days before. (H&E stain 100×).

Typical BCG induced lesions were observed in the footpad of mice treated with normal rat immunoglobulins (Fig. 5). The treatment of mice with immunoglobulins from immunized rats, induced diffuse necrosis of mononuclear cells in the granulomas, characterized by loss of cell sharp and nuclear picnosis. Phagocytosis of picnotic nuclei and cell debris by monocytoid macrophages (newly arrived cells) were observed (Fig. 6). This phenomenon was clearly characterized in five out of six examined lesions.

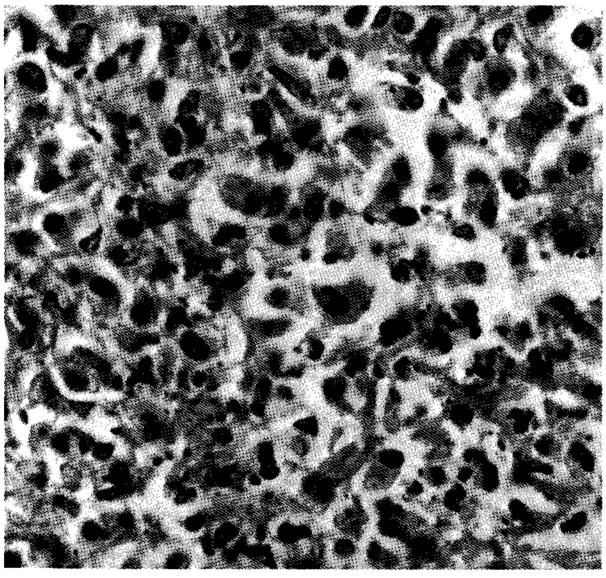

FIG. 6. Necrosis of inflammatory macrophages and phagocytosis of picnotic nuclei by newly arrived phagocytic mononuclear cells in granulomatous lesion induced by BCG inoculation 21 days before. The animals were treated for four consecutive days by intraperitoneal injection of immunoglobulins isolated from rats immunized with ECSS. (H&E stain 100×).

### Discussion

Mouse epithelioid macrophages are poor phagocytic cells;[4,14] they lose the capacity to release superoxide anion but secrete, among other not well characterized substances, an 11 kDa protein that deactivates activated macrophages and neutrophils.[8,9]

The origin of epithelioid cell secreted antigens was detected by immunocytochemical methods in cells removed after 14 but not after 5 days of coverslip implantation. The results show that although polyclonal, the antiserum raised against ECSS is able to discriminate epithelioid from other types of inflammatory macrophages. Further, the detection of similar epitopes in BCG induced granulomatas, demonstrate that these antigens are not restricted to the coverslip model but also are synthesized by cells in an experimental infectious lesion.

The detection of these antigens in cells on the coverslip or in cells from BCG induced granulomatas does not yield information concerning the possible role these substances play on the evolution, persistence and fate of the lesions. The significant decrease in the volume of the BCG induced granulomas, the marked necrosis of inflammatory cells and monocytoid cell infiltration of the lesions in animals injected with immunoglobulins from immunized rats are clear evidence that the blockage of antigens secreted by epithelioid cells drastically modifies the fate of this type of inflammatory process. The mechanisms by which these immunoglobulins induced this phenomenon was not determined. Nevertheless, Henriques *et al.*[15] have shown that 90% of inflammatory macrophages collected after 24 h of carrageen inoculation into the pleural cavity of mice, immunostain with the anti-ECSS antibodies. Yet, they have shown that these cells also secrete a factor which deactivates activated macrophages as does MDF. A polyclonal antibody directed to antigens secreted by macrophages from 24 h pleural carrageen induced lesions not only immunostained these cells but also cells on the surface of coverslips removed after 14 days, but not from 5 days after implantation in the subcutaneous tissue of mice. The inoculation of this antibody followed by carrageen injection into the pleural cavity of mice, completely abrogated the onset of cells expressing antigens revealed by antibodies anti-ECSS. These results provide evidence to support the suggestion that, similar to the necrosis of inflammatory cells observed in BCG induced granulomatas of mice treated with anti-ECSS, antibodies directed to secretory products of macrophages from carrageen induced pleurisy also kill or prevent the onset of cells which secrete MDF. In these experiments, after antibody treatment, almost all mononuclear adherent cells present in the pleural cavity were of the $Mac1^+$ phenotype or activated macrophages. This indirect evidence supports the hypothesis that antibodies anti-ECSS not only block MDF activity, as shown *in vitro*, but also may delete cells carrying antigens similar to those secreted by epithelioid cells.

The observed effect of the polyclonal antibodies directed to ECSS might be due to the inhibition of known mediators of granuloma formation and persistence. Kindler *et al.*[16] demonstrated that anti-TNF antibodies block the onset of granulomatas induced by BCG inoculation in mice. Based on their results, it is likely that anti-ECSS polyclonal antibody might have anti-TNF activity. This possibility was discarded since anti-$CCM_{14}$ antibodies do not block TNF necrotic activity on the L-929 cell line. TNF activity was also not detected in $CCM_{14}$ (data not shown).

Among other factors with MDF activity,[17–22] it has been demonstrated that IL-10, similarly to MDF,[8] also deactivates macrophages *in vitro* in a short period of contact[23] and that anti-IL-10 monoclonal antibody administration interferes with the outcome of *Trypanosoma cruzi* infection in mice.[24] To rule out the presence of IL-10 in $CCM_{14}$, a monoclonal antibody anti-IL-10 was added to $CCM_{14}$ and the deactivating property of this supernatant tested. The monoclonal antibody did not block the inhibitory activity of MDF as measured by superoxide anion release by BCG activated macrophages, thus indicating that MDF do not share epitopes similar to those of IL-10.

In conclusion, the marked effect of immunoglobulins isolated frcm anti-ECSS serum on the size and morphology of BCG induced granulomatas, demonstrates that substances secreted by epithelioid cells do play a role in the maintenance and fate of the lesion. As previously hypothesized[10] epithelioid cell secretory products such as MDF, are determinants of granuloma persistence by deactivating T-cell activated macrophages that migrate to maintain the cell turnover of the lesion and eliminate parasites.

### References

1. Adams DO. The structure of mononuclear phagocytes differentiation *in vivo*. 1. Sequential fine and histologic studies of the effect of bacillus Calmette–Guérin (BCG). *Am J Pathol* 1974; **76:** 617–648.
2. Adams DO. The granulomatous inflammatory response. *Am J Pathol* 1976; **84:** 164–191.
3. Spector WG, Mariano, M. Macrophage behaviour in experimental granulomas. In Van Furth R ed. *Mononuclear Phagocytes*. Oxford: Blackwell, 1975; 927–942.
4. Mariano M, Nikitin T, Malucelli BE. Immunological and non-immunological phagocytosis by inflammatory macrophages, epithelioid cells and macrophage polykarions from foreign body granulomata. *J Pathol* 1976; **120:** 151–159.
5. Mariano M, Malucelli BE. Defective phagocytic ability of epithelioid cells reversed by levamisole. *J Pathol* 1980; **130:** 33–36.
6. Papadimitriou JM, Spector WG. The origin, properties and fate of epithelioid cells. *J Pathol* 1971; **105:** 187–203.
7. Turk JL. Current status review: a comparison of secretory epithelioid cells and phagocytosing macrophages in experimental mycobacterial granulomas. *Br J Exp Pathol* 1989; **70:** 589–593.
8. Camarero VCPC, Junqueira V, Colepicolo P, Karnovsky ML, Mariano M. Epithelioid macrophages secrete a deactivating factor for superoxide release. *J Cell Physiol* 1990; **145:** 481–487.
9. Camarero VCPC, Junqueira V, Colepicolo P, Karnovsky ML. (Personal communication.)
10. Mariano M. Does macrophage deactivating factor play a role in the maintenance and fate of infectious granulomata? *Mem Instit Oswaldo Cruz* 1991; **86:** 485–487.
11. Ryan GB, Spector WG. Macrophage turnover in inflamed connective tissue. *Proc R Soc Lond* 1970; **B175:** 269–292.
12. Ove Nilsson B, Svalander PC, Larsson A. Immunization of mice and rabbits by intrasplenic deposition of nanogram quantities of protein attached to Sepharose beads or nitrocellulose paper strips. *J Immunol Methods* 1987; **99:** 67–75.
13. Johnston RB. Measurement of $O_2$-secreted by monocytes and macrophages. *Methods Enzymol* [46] 1984; **105:** 365–372.
14. Mariano M, Nikitin T, Malucelli BE. Phagocytic potential of macrophages from within delayed hypersensitivity-mediated granulomata. *J Pathol* 1977; **3:** 27–35.
15. Henriques MGMO, Monteiro MM, Vanni C, Pirmez C, Mariano M, Ribeiro–dos-Santos R. (submitted)
16. Kindler V, Sappino A, Grau GE, Piquet P, Vassalli P. The inducing role of tumor necrosis factor in the development of bacterial granulomas during BCG infection. *Cell* 1989; **56:** 731–740.
17. Ding A, Nathan CF, Graycar J, Derynck R, Stuehr DJ, Srimal M. Macrophage deactivating factor and transforming growth factors-§1, §2, and §3 inhibit induction of nitrogen oxide synthesis by IFN-$\gamma$. *J Immunol* 1990; **145:** 940–949.
18. Nong YH, Titus RG, Ribeiro JMC, Remold HG. Peptides encoded by the calcitonin gene inhibit macrophage function. *J Immunol* 1989; **143:** 45–52.
19. Szuro-Sudol A, Nathan CF. Supression of macrophage oxidative metabolism by products of malignant and non-malignant cells. *J Exp Med* 1982; **156:** 945–956.
20. Szuro-Sudol A, Murray HW, Nathan CF. Supression of macrophage oxidative metabolism by products of malignant and non-malignant cells. *J Immunol* 1983; **131:** 384–391.
21. Tsunawaki S, Nathan CF. Macrophage deactivation: altered kinetic properties of superoxide producing enzyme after exposure to tumour-cell conditioned medium. *J Exp Med* 1986; **164:** 1319–1331.
22. Tsunawaki S, Sporn M, Ding A, Nathan CF. Deactivation of macrophages by transforming growth factor-$\beta$. *Nature (Lond)* 1988; **334:** 260–265.
23. Bogdan C, Vodovotz Y, Nathan CF. Macrophage deactivation by interleukin 10. *J Exp Med* 1991; **174:** 1549–1554.
24. Silva JS, Morrissey PJ, Grabstein KH, Mohler KM, Anderson D, Reed SG. Interleukin 10 and interferon gamma regulation of experimental. *Trypanosoma cruzi* infection. *J Exp Med* 1992; **175:** 169–174.

ACKNOWLEDGEMENTS. The authors are indebted to Dr Nancy Starobinas for the TNF assays. Dr Ises Abrahamsohn kindly provided us with the anti-IL-10 monoclonal antibody. The technical assistance of Divaldo de Sousa is also acknowledged. This work was financially supported by grants from FAPESP and CNPq.

**Received 24 March 1993; accepted 1 April 1993**
